# Effect of low-magnitude, variable-frequency vibration therapy on pain threshold levels and mobility in adults with moderate knee osteoarthritis—randomized controlled trial

**DOI:** 10.1186/s12891-023-06334-9

**Published:** 2023-04-13

**Authors:** Alicja Pasterczyk-Szczurek, Joanna Golec, Edward Golec

**Affiliations:** 1 Vitberg Jacek Sikora, Nowy Sącz, Poland; 2 Academy of Physical Education in Krakow, Krakow, Poland

**Keywords:** Vibration, Physical therapy modalities, Osteoarthritis, Knee, Pain, VAS, ROM

## Abstract

**Background:**

Osteoarthritis (OA) is one of the most commonly recorded diseases in clinical practice. Vibration therapy has been suggested for the treatment of knee OA. The purpose of the study was to determine the impact of vibrations of variable frequency and low amplitude on pain perception and mobility in patients suffering from knee OA.

**Methods:**

Thirty-two participants were allocated into two groups – Group 1 (oscillatory cycloidal vibrotherapy-OCV) and Group 2—control (sham therapy). The participants were diagnosed with moderate degenerative changes in the knee (grade II based on the Kellgren-Lawrence (KL) Grading Scale). Subjects received 15 sessions of vibration therapy and sham therapy respectively. Pain, range of motion, and functional disability were assessed through Visual Analog Scale (VAS), Laitinen questionnaire, goniometer (ROM – range of motion), timed up and go test (TUG) and Knee Injury and Osteoarthritis Outcome Score (KOOS). Measurements were taken at baseline, after the last session and four weeks after the last session (follow up). T-test and U-Mann Whitney test compare baseline characteristics. The Wilcoxon and ANOVA tests compared mean VAS, Laitinen, ROM, TUG and KOOS. The significant *P*-value was less than 0.05.

**Results:**

After 3 weeks (15 sessions) of vibration therapy, reduced the sensation of pain and improved mobility was recorded. There was a more significant improvement in the vibration therapy group than the control group in pain alleviation on VAS scale (*p* < 0.001), on Laitinen scale (*p* < 0.001), knee ROMs flexions (*p* < 0.001) and TUG (*p* < 0.001) at the last session. KOOS score with pain indicator, symptoms, activities of daily living, function in sport and recreation and knee related quality of life improved more in the vibration therapy group than the control group. Effects maintained up to 4 weeks in vibration group. No adverse events were reported.

**Conclusions:**

Our data demonstrated that the use of vibrations of variable frequency and low amplitude in patients with the knee OA is a safe and effective therapy. It is recommended to increase the number of treatments performed, primarily in patients with degeneration II° according to the KL classification.

**Trial registration:**

Prospectively registered on ANZCTR (ACTRN12619000832178). Registered on 11 June 2019.

## Background

Osteoarthritis (OA) is one of the most frequently recorded diseases in clinical practice [1]*.* It is the result of overlapping diseases of various kinds, which, despite their different aetiology, lead to analogous biological, morphological and clinical effects [2]. It is defined as one of the components of the natural ageing process of the body, resulting from the mechanical wear (abrasion) of the joint surfaces during physical activity. Therefore, age is one of the risk factors for osteoarthritis [3–6]. The main cause of these changes is the imbalance between the processes of degeneration and regeneration of the articular cartilage and the subcartilage layer*,* including those which largely depend on enzymatic activity [7, 8]*.* These changes usually develop slowly and, worsening over time, inevitably lead to painful limitation of joint mobility, deformation and contracture, and limb axis distortion*.* It is registered more often in women than in men, and above all, after the age of 50. In people over 65 years of age, its morphology, course and clinical expression are also significantly influenced by involutional degenerative and hormonal changes that develop over time in the osteoarticular and nervous systems [5–7]*.* One of the many problems with osteoarthritis is the sensation of pain. Initially, it is associated with physical exertion (post-exercise pain), and with time it takes the form of night and rest symptoms [9]*.* It is also associated with posturographic and stabilometric changes [10–12]*.* The result is limited physical activity, which makes further treatment difficult. Vibrotherapy is indicated as one of the treatment regimens for OA. Such therapy is defined as a method of effective, non-pharmacological and inoperable treatment of patients with osteoarthritis of knee joints [13]. In the method, constant frequencies which are designed to improve the overall functioning of the muscular-ligament and joint apparatus are most often used locally [14]. Pamukoff et al. indicated. that the action of Local Muscle Vibration (LMV) and the Whole Body Vibration (WBV) significantly improved the function of the quadriceps muscle and may be useful methods of restoring its strength in people with degenerative changes of the knee joints [15]. Vibrotherapy in the treatment of degenerative joint changes is the method which is more and more often used both in the area of the whole body and locally at the site of the disease [14–16].

## Methods

The aim of the study was to determine the effectiveness of vibratory stimulation in supine position on pain thresholds level, range of motion, and functional disability in OA patients. The study was conducted to summarize and determine the efficacy of vibration therapy of individuals with osteoarthritis. Standing position is not easy to manage for all patients. Therefore, there is a need for further studies of the effects of local vibrations in a free-position system as a non-pharmacologic and non-invasive therapy. It was a single-blinded, controlled, two-group parallel design. Patients were allocated to either vibration therapy group or control group. The experimental subjects were osteoarthritis volunteers recruited from the physiotherapy clinics. They were informed of the purpose of the experimental procedures before the experiments commenced and that they could leave at any time. The study was approved by the Ethical Committee of Regional Medical Chamber in Cracow [Ref no. 120/KBL/OIL/2018].

### Study population

The study involved 32 people in total, divided into two groups. The sample size was determined by performing a priori power analysis. 16 patients were included in the treatment group. The control group also included 16 people. The age of those included in the study ranged from 42 to 79 years (mean 64.28 years), their mean body weight was 82.18 kg, and their body height was 1.62 m. Advanced degenerative changes of the knee joints of the grade II were diagnosed in the patients included in the study based on the Kellgren-Lawrence scale [17]. The advancement of the degeneration was assessed by a specialist in the field of orthopaedics and traumatology of the musculoskeletal system. The knee joint, which was characterized by a higher degree of advancement of degenerative changes, was taken into account for the analysis.

### Study duration

The duration of the study was from January 2019 to May 2021.

### Data collection procedure

The data collection was started after the approval from Institutional Review Board, Academy of Physical Education in Cracow. The recruitment of patients to participate in the presented studies lasted from 2019 to 2021 (from medical facilities in Nowy Sącz, Poland). Participants were assigned to the random numbers from random number tables to the treatment conditions. The block size was determined by the researcher and was multiple with two treatment groups. Thirty-two patients enrolled in the study were divided into those who underwent therapy (the group that underwent vibrotherapy treatments) and those that were the control group (sham treatments). The study included people with no contraindications to vibrotherapy in accordance with the Instructions for Use of the RAM Vitberg + vibrotherapy apparatus (Vitberg + Rehabilitation Massage Unit; Vitberg, Poland, previous generation of Vitberg Recovery System models); diagnosed with degenerative disease of the grade II according to the Kellgren-Lawrence scale with reference to at least one knee joint, lasting at least 4 years. The following inclusion and exclusion criteria were adopted for the presented studies: age between 40 and 85; no contraindications to vibrotherapy in accordance with the RAM Vitberg + Instructions for Use (aneurysms, venous thrombosis, atherosclerosis, conditions after recent myocardial infarctions and strokes, severe inflammation, epilepsy, diseases with dizziness, intellectual limitations and mental performance, recent traumatic injuries to tendons, ligaments and muscles, chronic kidney and gallbladder stones, hemorrhages, active cancer processes, increased body temperature, skin wounds, pregnancy and the postpartum period), no sensory disturbances in the area of ​​examination; no venous thrombosis or post-thrombotic syndrome; lack of neurological diseases, including those resulting in paresis or hemiplegia; no malignant disease. The recruitment to the proposed study was based on medical qualifications, including available medical documentation (medical history, information sheets, outpatient treatment cards, including physiotherapy treatment cards). During the study, participants were asked not to change their eating habits, take the medications administered so far, not to change their current physical (recreational) activities, not to use any physiotherapeutic procedures except those administered during the study. The order of applications and the availability of patients determined the assignment to a group in a given round. The data was collected through questionnaires and measurements before initiating the first physiotherapy session for baseline comparison of both groups and after the last physiotherapy session. Then 4 weeks after the last session to get information about follow up period. The whole procedure for assessing outcomes has taken 8–16 min.

### Masking

This study was a single-blinded controlled trial in which the patients were prevented from knowing the interventions assigned to them.

### Intervention

**Fig. 1:**
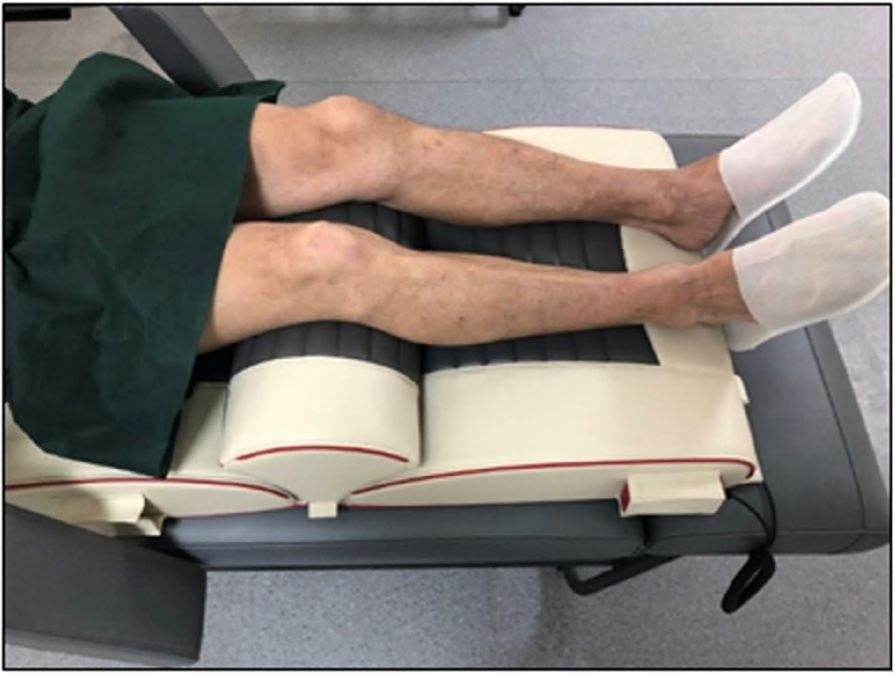
Position during treatments

Vibration therapy was provided by the medical device—RAM Vitberg + Base Module and RAM Vitberg + Knee Module (previous series of Vitberg Recovery System medical device) (Fig. 1). The frequency of the vibration varied over time (5–50 Hz), low amplitude (0–0.2 mm) with a peak-to-peak acceleration of 1,38 g (g = gravitational acceleration). This type of vibrotherapy is referred to by the manufacturer as Oscillatory Cycloid Vibrotherapy (OCV). The subjects participated in a 3-week cycle of vibrotherapy or sham therapy, lasting ~ 60 min each, in a semi-recumbent position. Vibrotherapy treatments were performed in the number of 15, divided into 3 series of treatments, for 3 weeks (5 treatments during the next 5 treatment days), once a day and covered both knee joints. The devices for sham therapy did not have the function of generating measurable vibrations (acceleration a < 0.01 m / s2; frequency f < 0.01 Hz, amplitude A < 0.01 mm). From the outside, the devices did not differ visually from the vibration therapy devices and gave the same sound and light signals at the beginning and end of the therapy. The study was not blinded, and the researchers were aware of which stages of the study had been carried out on devices with vibrotherapy and which on sham devices.

### Outcome measures

The primary outcome measures used in this study were VAS [18] and Laitinen Questionnaire for pain and secondary outcome measures were TUG (Timed Up and Go Test) [19], active range of motion [20] and KOOS measurements [21].

### Visual analog scale

The evaluation of knee joint pain was based on the Visual Analogue Scale (VAS) [18]. VAS is a widely accepted method of measuring the degree of pain and has been defined as a sensitive and reliable method compared to others [22]. VAS allows the patient to determine the pain level ranging from 0 to 10, with 0 being no pain at all and 10 the maximum (imaginable). Patients marked the level of pain immediately before and immediately after the procedures.

### Laitinen questionnaire

The Laitinen Pain Questionnaire is a subjective and point-based tool used to assess the level of pain symptoms [23]. Each indicator is assigned points from 0 to 4, where 0 is no problem and 4 is maximum problem. Bipolar 5-step gradation is used. The total assessment of pain according to the Laitinen Pain Questionnaire is the sum of points from four groups, i.e. pain intensity, pain frequency, frequency of taking painkillers and limitation of physical activity. The maximum number of points that can be obtained is 16—which means an advanced level of pain, and 0 means no pain. Patients assessed the level of pain immediately before and immediately after the procedures.

### Active range of motion

The range of active movement in the knee joint was measured. The patient was lying on the couch in the forward lying position. The test was performed with the company's goniometer in accordance with the accepted standards [20]. All research tools used in the study were certified.

### TUG test

The functional state of the knee joints was tested in a fixed time schedule. The study included the subject assessment through the TUG test (Timed Up and Go Test). The test was carried out so that the patient was properly positioned on the test stand. The patient sat on a stable chair. After declaring readiness, the task was to get up, cover a distance of 3 m and return to the chair. The test was carried out in triplicate and the mean was calculated. Normal value for a healthy adult is 10 s (s) or less [19].

### KOOS questionnaire

KOOS (Knee Injury and Osteoarthritis Outcome Score) is used to assess the functional status of OA patients. The respondents indicated one answer that described the symptoms that had occurred during the last week. The questionnaire consists of 5 parts. In the first part, the patient determines the characteristics of the pain. In the second, he/she determines the severity of symptoms. In the third part, the patient defines the degree of difficulty in performing typical daily activities. The fourth part concerns sports and recreational activities, and the fifth part concerns the quality of life [9, 21].

### Harm and adverse events

There were no occurrences of harm and adverse event reported during the period of trial.

### Statistical analysis

The statistical analysis was done using Statistica version 13.0 software for Windows (Statsoft). All data are expressed as mean value ($\underline{x}$), standard deviation (SD), percentage distribution and 95% confidence intervals (95% CI). When analyzing the normal distribution, in order to compare the results obtained in the measurements before the treatments, the Student's t-test was performed for independent samples. When analyzing variables that were not normally distributed, the Mann–Whitney U test was used to compare two independent samples (results between groups). For individual measurement points of normally distributed variables, ANOVA was used with repeated measurements for dependent variables, with a qualitative factor grouping into the treatment group (Group 1) and control (Group 2). The Wilcoxon test was performed for pairs of observations in individual populations. Significant differences were determined at *P* < 0.05.

## Results

Characteristics of 32 patients are presented in Table 1. The mean age of patients was 64.28 ± 9.23.

**Table 1:** Characteristics of study patients (*n* = 32)

**Characteristics**^**a**^	**Group 1 (Vibration- OCV therapy) *n* = 16**	**Group 2 (Control)- sham therapy *n* = 16**	**Total *n* = 32**	*p*-value*
**Age (years)**	63.69 ± 7.61	64.88 ± 10.84	64.28 ± 9.23	0.72
**Height (meters)**	1.62 ± 0.83	1.63 ± 0.86	1.62 ± 0.83	0.68
**Weight (kg)**	81.34 ± 17.71	83.02 ± 15.50	82.18 ± 16.39	0.78
**BMI (kg/m2)**	31.04 ± 5.63	31.36 ± 5.51	31.20 ± 5.48	0.87
**Gender:**				
** Male**	1 (20.00%)	4 (80.00%)	5 (15.6%)	
** Female**	15 (55.56%)	12 (44.44%)	27 (84.4%)	

Values presented as level of significance with independent T test

Values represented as mean ($\underline{x}$) and standard deviation (SD) or frequency (percentage)

The mean height was 1.62 ± 0.83 m. The frequency and percentage of female patients were higher than the male patients. The mean BMI of patients was 31.20 ± 5.48.

In the presented studies, it was observed that the use of OCV in patients with knee OA reduces the perception of pain. In Table 2, Group 1 (OCV therapy) shows more significant (< 0.001) improvement in pain level as compared to Group 2 (sham therapy) at the 1st and at the last session and at the 1st session and 4 weeks after the last session. The VAS scores improved more by 2.06 (< 0.001) in the Group 1 than the Group 2, which improved by 0.63 (> 0.05). For weeks after (follow up measurement) in Group 1 the level of pain remained the same level (Table 2).

**Table 2:** Between-group and within-group comparisons of VAS (*N* = 32)

**Groups *n* = 32**	**Baseline**	**The last session** **(15**^**th**^**session)**	**Follow up**	**Within-group comparison**^**b**^	
				**Baseline vs 15**^**th**^** session**	**Baseline vs follow up**
**Group 1 (*n* = 16)**^a^	5.44 ± 1.46 (1.08–2.26)	3.38 ± 1.36 (1.00–2.11)	3.38 ± 1.31 (0.97–2.03)	2.06 (< 0.001)	2.06 (< 0.001)
**Group 2 (*n* = 16)**^a^	4.88 ± 1.31 (0.97–2.03)	4.25 ± 1.00 (0.74–1.55)	3.88 ± 0.72 (0.53–1.11)	0.63 (> 0.05)	3.56 (< 0.02)
**Between-group comparison**^**b**^ **(*p*-value)**	0.56 (> 0.05)	-0.87 (< 0.05)	-0.50 (> 0.05)		

^a^Values presented as mean ± standard deviation (95% CI);

^b^Values presented as mean difference

In Table 3, Group 1 (OCV) shows more improvement in Laitinen scores at both the last session and the follow up as compared to Group 2 (ST). Mean Laitinen scores for the OCV group were 3.81 ± 1.56 at the final session compared to the ST group, 4.19 ± 1.28 (Table 3).

**Table 3:** Between-group and within-group comparisons of Laitinen (*N* = 32)

**Groups** ***n* = 32**	**Baseline**	**The last session** **(15**^**th**^** session)**	**Follow up**	**Within-group comparison**^**b**^	
				**Baseline vs the last session**	**Baseline vs follow up**
**Group 1 (*n* = 16)**^a^	6.06 ± 2.05 (1.51–3.17)	3.81 ± 1.56 (1.15–2.41)	3.88 ± 2.31 (1.70–3.57)	2.25 (< 0.002)	2.18 (< 0.009)
**Group 2 (*n* = 16)**^a^	5.44 ± 1.86 (1.37–2.88)	4.06 ± 1.65 (1.22–2.56)	4.19 ± 1.28 (0.94–1.98)	1.38 (< 0.04)	1.25 (< 0.04)
**Between-group comparison**^**b**^ **(*p*-value)**	2.18 (> 0.05)	-4.47 (> 0.05)	-0.11 (> 0.05)		

^a^Values presented as mean ± standard deviation (95% CI)

^b^Values presented as mean difference

In Table 4, knee ROMs improved significantly (< 0.01) in Group 1 (OCV) as compared to Group 2 (ST) in the last session from baseline. There was more substantial improvement than baseline in flexion ranges after 15th session in OCV group (Table 4).

**Table 4:** Between-group and within-group comparisons of ROM (*N* = 32)

**Groups *n* = 32**	**Baseline**	**The last session** **(15**^**th**^** session)**	**Follow up**	**Within-group comparison**^**b**^	
				**Baseline vs the last session**	**Baseline vs follow up**
**Group 1 (*n* = 16)**^a^	102.94 ± 24.48 (18.09–37.89)	108.88 ± 19.05 (14.07–29.48)	107.00 ± 16.47 (12.17–25.49)	5.94 (< 0.01)	4.06 (> 0.05)
**Group 2 (*n* = 16)**^a^	107.50 ± 17.03 (12.58–26.36)	107.19 ± 14.94 (11.04–23.12)	108.13 ± 12.63 (9.33–19.55)	-0.31 (> 0.05)	0.63 (> 0.05)
**Between-group comparison**^**b **^**(*p*-value)**	-4.56 (> 0.05)	1.69 (> 0.05)	-1.13 (> 0.05)		

^a^Values presented as mean ± standard deviation (95% CI)

^b^Values presented as mean difference

In Table 5, TUG improved significantly (< 0.01) in Group 1 (OCV) as compared to Group 2 (ST) in the last session from baseline and even more at follow up (< 0.001). There was more substantial improvement than baseline after 15th session in OCV group (Table 5).

**Table 5:** Between-group and within-group comparisons of TUG (*N* = 73)

**Groups *n* = 32**	**Baseline**	**The last session** **(15**^**th**^** session)**	**Follow up**	**Within-group comparison**^**b**^	
				**Baseline vs the last session**	**Baseline vs follow up**
**Group 1 (*n* = 16)**^a^	10.60 ± 2.75 (2.03–4.25)	8.99 ± 2.25 (1.66–3.49)	8.50 ± 1.36 (1.01–2.11)	1.61 (< 0.01)	2.10 (< 0.001)
**Group 2 (*n* = 16)**^a^	11.45 ± 2.58 (1.91–4.00)	10.92 ± 3.53 (2.61–5.46)	10.15 ± 3.71 (2.74–5.74)	0.53 (> 0.05)	1.30 (< 0.05)
**Between-group comparison**^**b **^**(*p*-value)**	-0.85 (> 0.05)	-1.93 (< 0.02)	-1.65 (> 0.05)		

^a^Values presented as mean ± standard deviation (95% CI)

^b^Values presented as mean difference

In Table 6, KOOS indicators improved significantly at pain scores (< 0.004) in Group 1 (OCV) as compared to Group 2 (ST) in the last session from baseline and at follow up (< 0.03). There was more substantial improvement than baseline after 15th session in OCV group. Symptoms of the OA improved significantly only in group 1 (OCV) after the last session (< 0.005) and at follow up (< 0.006). Activities of daily living (ADL) improved more in in group 1 (OCV) after the last session (< 0.005) and at follow up (< 0.005). Also, sport and recreation function improved more in in group 1 (OCV) then in group 2 (ST). Quality of life improved significantly only in group 1 (OCV) after the last session (< 0.003) and at follow up (< 0.005) (Table 6).

**Table 6:** Between-group and within-group comparisons of KOOS (*N* = 32)

**Groups *n* = 32**	**Baseline**	**The last session** **(15**^**th**^** session)**	**Follow up**	**Within-group comparison**^**b**^	
				**Baseline vs the last session**	**Baseline vs follow up**
**Pain:**^a^					
** Group 1 (*n* = 16)**	55.54 ± 18.84 (13.92–29.16)	67.04 ± 14.57 (10.76–22.54)	66.42 ± 17.84 (13.18–27.61)	11.5 (< 0.004)	10.88 (< 0.03)
** Group 2 (*n* = 16)**	56.55 ± 15.39 (11.37–3.82)	64.77 ± 15.46 (11.42–23.93)	61.08 ± 15.27 (11.28–23.63)	8.22 (> 0.05)	4.53 (> 0.05)
** Between-group comparison**^**b **^**(*p*-value)**	-1.01 (> 0.05)	2,27 (> 0.05)	5,34 (> 0.05)		
**Symptoms:**^a^					
** Group 1 (n = 16)**	51.66 ± 21.76 (16.07–33.68)	66.11 ± 19.50 (14.41–30.18)	64.84 ± 24.09 (17.80–37.29)	14.45 (< 0.005)	13.18 (< 0.006)
** Group 2 (n = 16)**	52.34 ± 21.87 (16.15–33.84)	60.13 ± 16.81 (12.42–26.02)	56.76 ± 15.63 (11.55–24.19)	7.79 (> 0.05)	4.42 (> 0.05)
** Between-group comparison**^**b **^**(*p*-value)**	-0.68 (> 0.05)	5.98 (> 0.05)	8.08 (> 0.05)		
**ADL:**^a^					
** Group 1 (n = 16)**	50.12 ± 20.96 (5.48–32.44)	68.26 ± 16.70 (12.34–25.85)	68.80 ± 16.71 (12.34–25.86)	18.14 (< 0.005)	18.68 (< 0.005)
** Group 2 (n = 16)**	49.31 ± 13.14 (9.71–20.34)	60.24 ± 19.82 (14.64–30.67)	62.31 ± 16.24 (11.99–25.13)	10.93 (< 0.04)	13.00 (< 0.03)
** Between-group comparison**^**b **^**(*p*-value)**	0,99 (> 0.05)	8,02 (> 0.05)	6.49 (> 0.05)		
**Sport/Rec:a**					
** Group 1 (n = 16)**	35.47 ± 24.70 (18.24–38.22)	49.84 ± 25.52 (18.85–39.49)	49.19 ± 30.36 (22.43–46.99)	14.37 (> 0.05)	13.72 (< 0.05)
** Group 2 (n = 16)**	34.67 ± 16.40 (12.12–25.38)	39.61 ± 22.05 (16.29–34.13)	39.30 ± 20.84 (15.39–32.25)	4.94 (> 0.05)	-0.31 (> 0.05)
**Between-group comparison**^**b **^**(*p*-value)**	0,80 (> 0.05)	10,23 (> 0.05)	9,89 (> 0.05)		
**QOL:**^a^					
** Group 1 (n = 16)**	33.60 ± 16.64 (12.30–25.76)	46.74 ± 16.63 (12.29–25.74)	49.22 ± 21.27 (15.71–32.92)	13.14 (< 0.003)	15.62 (< 0.005)
** Group 2 (n = 16)**	40.49 ± 10.92 (8.06–16.90)	46.09 ± 15.12 (11.17–23.40)	45.70 ± 13.45 (9.93–20.81)	5.60 (> 0.05)	5.21 (> 0.05)
** Between-group comparison**^**b **^**(*p*-value)**	-6.89 (> 0.05)	0.65 (> 0.05)	3.52 (> 0.05)		

^a^Values presented as mean ± standard deviation (95% CI)

^b^Values presented as mean difference

## Discussion

The current study focused on the effects of OCV vibrotherapy on the level of symptoms associated with knee osteoarthritis. The aim of the study was to compare the effects of OCV vibrotherapy with sham therapy in reducing pain and functional disability in people with knee osteoarthritis. The study showed a significant improvement in the group that received OCV, as the appropriate vibration ranges are able to stimulate the body to regenerate [24–26]. Recently, therapeutic vibrations in the form of vibrotherapy have been included in the physiotherapy of osteoarthritis [27]*.* Vibrotherapy is also combined with other methods. including, inter alia, local or systemic administration of substances inhibiting inflammation (non-steroidal and steroidal anti-inflammatory drugs) [16].

According to the authors, the study's most important finding was non-pharmacological pain relief. Pain relief immediately after vibrotherapy is explained by the complex theory of pain [28–34]. In the present study, the VAS and Laitinen scores indicate an analgesic effect of vibrotherapy that may last for 4 weeks. Lurie et al. [35] and McGinnis et al. [36] found similar results in terms of efficiency and safety. Lurie et al., studied patients in whom he identified low back pain (LBP). He reported statistically lower VAS scores compared to the period before vibrotherapy. However, once the vibration stopped, the LBP returned to pre-vibration levels. This is inconsistent with the results of the trial. The effects of the Lurie study were only temporary. It is suggested that this may be due to the short duration of the therapy (3 min) [35]. On the other hand, McGinnis et al. [36] found that objects in the vibration group had significantly lower levels of pain and a more stable heart rate during the heel lance procedure and 2 min after the heel strike than those in the non-vibration group. No adverse behavioural or physiological responses to the applied vibration were observed in the sample. Similar to the conducted study—Guieu et al. found that 30 min of vibrotherapy in patients with chronic pain, has an analgesic effects [37].

The ROM measurement was used to test the mobility of the knee joints. The range of motion in flexion increased significantly in the group with vibrotherapy. The effect, however, was not sustained when measured after 4 weeks. Thanks to the cumulative analgesic effect, the reduction of inflammation and tissue swelling, the perceived stiffness of tissues, including joints, can be reduced. This is confirmed by Johnson et al. [38] and Peer et al. [39]. They demonstrate that vibrotherapy provides significant and measurable benefits in improving flexibility and reducing perceived stiffness in joints in patients with various types of traumatic musculoskeletal injuries. The effect of vibrotherapy on muscles can trigger the activity of fibroblasts and stimulate the correct activity of collagen fibres, allowing tissues to regain flexibility when stretched after traumatic injuries [39].

The research presented shows a significant improvement in the quality of life and functional status of patients with knee joint degeneration after using vibrotherapy. This is highlighted by the reduced TUG time. A reduction in TUG time was also observed in the control group. It is possible that this was caused by the daily physical activity of the patients who had to attend the sessions. The active ranges of motion in knee flexion increased during therapy, but this effect was not maintained satisfactorily over time after the end of treatment (in post-treatment measurements). Pamukoff et al. [15] showed that the effect of vibration applied locally and to the whole body significantly improves the function of the quadriceps muscle of the thigh and can be effective in restoring its strength in people with degenerative changes in the knee joints. Therefore, similar to the study, it was concluded that vibratory stimulation may be an appropriate intervention to acutely increase quadriceps function and may be useful to aid in the recovery of quadriceps strength in individuals with knee pathology. This is also evidenced by the studies conducted by Germann et al. [40] and Alam et al. who showed that vibrotherapy is the most effective when used in conditions of complete relaxation of the muscle tone. The tonic vibration reflex (TVR) activated this way stimulates involuntary muscle contractions forcing them to work without the participation and awareness of the patient [40–43]. Improvement in quality indicators reflecting functional status was seen in both study groups. However, the improvement in the vibrotherapy group was more pronounced and sustained. This may be due to the effect of increasing muscle strength after the treatments.

The KOOS adequately reflects treatment endpoints as it is used to assess short and long term symptoms and function in patients with knee injuries and osteoarthritis [44]. In the conducted studies, the group subjected to treatment had significantly better results in the subscales describing pain (Pain), symptoms (Symptoms), activities of daily living (ADL), activity in sport and recreation (Sport / Rec) and in the subscale of quality of life related to the knee (QoL). The results are in line with the findings of Paolucci et al. [45]. Patients with chronic pain (> 6 weeks) due to osteoarthritis of the knee joint (Kellgren-Lawrence grade II-III) were enrolled sequentially and divided into two groups: with intra-articular oxygen-ozone therapy (O2O3) and with O2O3 and vibrotherapy. VAS, KOOS and the Medical Research Council (MRC) Manual Muscle Testing Scale were assessed at baseline (pre-treatment), after 3 weeks of treatment and 1 month after the end of treatment. Patients received intra-articular injections of O2O3 into the knee joint three times a week. The vibrotherapy group also underwent nine sessions (three sessions per week). The results of the VAS, KOOS and MRC were significantly better in the O2O3 combined with vibrotherapy group than in the O2O3 alone group. Intra-group analysis showed that all scores improved over time from baseline and were maintained up to 1 month after treatment. No adverse events were reported during treatment.

As a result of the research carried out, it has been established that the improvement of medical technologies that enable non-invasive pain relief and the functional state of patients with degenerative knee joints can significantly contribute to accelerate the treatment process and reducing its costs in comparison with the traditional treatments for the lesions in question. Adapting vibrotherapy to the patient's age, sex, general and local condition, as well as checking the effectiveness of the treatment used to date, can significantly reduce the symptoms associated with OA. In many cases, the recommended physiotherapy cannot be carried out with the required frequency. This is especially true for patients with significant functional limitations and difficulties in reaching the place of treatment, which, according to Alami et al., is often underestimated by physicians [41]. Therefore, the use of therapeutic methods (e.g. vibrotherapy) that do not interfere with other methods of treating degenerative changes in the joints, including those of the knee, and that can be performed in the patient's home, can be an effective addition to the overall treatment process. Further research is needed into the repeated use of vibrotherapy and its ability to enhance the recovery process. However, the acute effects of this treatment in reducing pain and stiffness in OA injuries show potential as a therapeutic intervention.

### Limitations

The findings of this study have to be seen in the light of the following major limitations. This study recruited the patients with non-probability purposive sampling technique due to the nature of patients’ characteristics requiring rehabilitation.

## Conclusion

The study showed that vibrotherapy (OCV—Oscillatory Cycloid Vibrotherapy) is effective in relieving pain, improving range of motion and functional status in patients with OA. The therapy showed a significant improvement in terms of immediate and long-term influence on all analysed indicators. Therefore, it should be pointed out that it is an effective and safe method that can be added to conventional OA therapy to improve treatment outcomes, especially in patients with grade II OA. No adverse events occur.

## Data Availability

The datasets used and/or analysed during the current study are available from the corresponding author on reasonable request.

## Abbreviations

OA: Osteoarthritis
VAS: Visual Analog Scale
KOOS: Knee Injury and Osteoarthritis Outcome Score
WBV: Whole-body vibration
OCV: Oscillatory Cycloidal Vibrotherapy
ST: Sham Therapy
TVR: Tonic Vibration Reflex
